# The Role of Oxidative Stress, Mitochondrial Function, and Autophagy in Diabetic Polyneuropathy

**DOI:** 10.1155/2017/1673081

**Published:** 2017-10-24

**Authors:** Sonia Sifuentes-Franco, Fermín Paul Pacheco-Moisés, Adolfo Daniel Rodríguez-Carrizalez, Alejandra Guillermina Miranda-Díaz

**Affiliations:** ^1^Institute of Experimental and Clinical Therapeutics, Department of Physiology, University Health Sciences Centre, University of Guadalajara, Guadalajara, JAL, Mexico; ^2^Department of Chemistry, University Centre for Exact and Engineering Sciences, University of Guadalajara, Guadalajara, JAL, Mexico

## Abstract

Diabetic polyneuropathy (DPN) is the most frequent and prevalent chronic complication of diabetes mellitus (DM). The state of persistent hyperglycemia leads to an increase in the production of cytosolic and mitochondrial reactive oxygen species (ROS) and favors deregulation of the antioxidant defenses that are capable of activating diverse metabolic pathways which trigger the presence of nitro-oxidative stress (NOS) and endoplasmic reticulum stress. Hyperglycemia provokes the appearance of micro- and macrovascular complications and favors oxidative damage to the macromolecules (lipids, carbohydrates, and proteins) with an increase in products that damage the DNA. Hyperglycemia produces mitochondrial dysfunction with deregulation between mitochondrial fission/fusion and regulatory factors. Mitochondrial fission appears early in diabetic neuropathy with the ability to facilitate mitochondrial fragmentation. Autophagy is a catabolic process induced by oxidative stress that involves the formation of vesicles by the lysosomes. Autophagy protects cells from diverse stress factors and routine deterioration. Clarification of the mechanisms involved in the appearance of complications in DM will facilitate the selection of specific therapeutic options based on the mechanisms involved in the metabolic pathways affected. Nowadays, the antioxidant agents consumed exogenously form an adjuvant therapeutic alternative in chronic degenerative metabolic diseases, such as DM.

## 1. Introduction

Distal sensorimotor polyneuropathy is considered the most frequent diabetic polyneuropathy (DPN) and is the most prevalent chronic complication of diabetes mellitus (DM) [1]. It is possible that DPN is present in 10% of patients with an initial diagnosis of type 2 DM. In fact, emerging data suggest that DPN can occur before the development of hyperglycemia in the diabetic range in people with metabolic syndrome or altered tolerance to glucose [2]. The DPN can affect ~50% of patients with long-term DM [3]. The prevalence of DPN increases with age and history of the disease and is typically characterized by deficient control of glycemia [4]. The objective of the present review was to describe the mechanisms of functional and structural damage in DPN, the role and participation of oxidative stress, the oxidative stress of the endoplasmic reticulum, the behavior of the antioxidants, the effect on mitochondrial function, and autophagy in DPN.

## 2. Functional and Structural Damage to the Nervous Tissue in DPN

**Figure 1:**
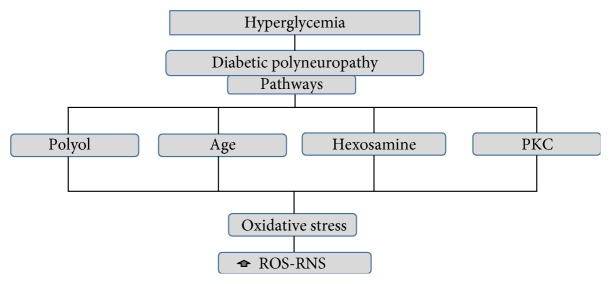
Interaction of hyperglycemia pathways with oxidative stress in DPN.

The potential mechanisms that lead to the damage of the nervous tissue in DPN include the activation of the different pathways: (a) the polyol pathway (glucose metabolism), (b) the deposit of end-products of advanced glycosylation, (c) the poly(ADP-ribose) polymerase, (d) the hexosamine pathway, and (e) the protein kinase C pathway. All these pathways are activated in the state of hyperglycemia (Figure 1()). Each one of the pathways has the capacity to produce vascular insufficiency and oxidative stress [5]. The hyperglycemic state triggers the increase in the production of mitochondrial and cytoplasmic ROS, which, in conjunction with the deregulation of the antioxidant defenses, activates new pathways capable of producing oxidative damage in DPN [6, 7].

## 3. Oxidative Stress

Free radicals such as hydroxyl radical (HO•), nitric oxide (•NO), peroxynitrite (ONOO−), superoxide anion (O_2_•−), nitrogen dioxide (•NO_2_), peroxyl radicals (ROO•), and lipid peroxyl (LOO•) are highly reactive, unstable molecules that have an unpaired electron in their outer shell. ROS comprehends free radical and nonradical molecules. Nonradicals include hydrogen peroxide (H_2_O_2_), singlet oxygen (^1^O_2_), and lipid peroxide (LOOH), among others. H_2_O_2_ is a major ROS in cells and can diffuse long distances crossing membranes and causing cell damage at high concentrations by reacting with transition metals (copper, iron (Fe), and cobalt) yielding HO• via the Fenton reaction [8]:

(1) $$\begin{array}{c} {Fe}^{++}+H_{2}O_{2}\to {Fe}^{+++}+{HO}^{–}+HO• \end{array}$$

ROS and reactive nitrogen species (RNS) are formed during normal metabolic activity in a variety of biochemical reactions and cellular function. Their beneficial effects occur at low concentrations and involve physiological roles in cellular signaling systems. For example, H_2_O_2_ is produced in response to cytokines and growth factors and is involved in regulating immune cell activation and vascular remodeling in mammals [9]. NO• is generated *in vivo* by specific NO synthases (NOS) and the nitrate-nitrite-NO pathway and is a critical regulator of vascular homeostasis, neurotransmission, and host defense [10]. Excessive NO• production, under pathological conditions, leads to detrimental effects of this molecule on tissues, which can be attributed to its reaction with superoxide anion (O_2_•−) to form ONOO−. ONOO− is 1000 times more potent as an oxidizing compound than H_2_O_2_ [11].

The main sources of ROS are the mitochondrial electron transport chain and enzymatic reactions catalyzed by NOS, NADPH oxidases, xanthine oxidase, and hemeperoxidase enzymes, such as myeloperoxidase. The nonenzymatic production of O_2_•− occurs when a single electron is directly transferred to oxygen by reduced coenzymes or prosthetic groups (Flavin's or iron sulfur clusters) or by xenobiotics previously reduced. Ubisemiquinone autoxidation (ubisemiquinone donates one electron to molecular oxygen yielding O_2_•− and ubiquinone) is the major source of O_2_•− in mitochondria, and because the ubiquinone or coenzyme Q pool faces both the intermembrane space and the mitochondrial matrix, O_2_•− is vectorially released into both compartments. O_2_•− released in the intermembrane space can cross the outer mitochondrial membrane into the cytosol through the porin protein. Furthermore, the mitochondrial electron transport chain contains several redox centers that may leak electrons to oxygen [12].

Under physiological conditions, the steady-state formation of ROS and RNS is normally balanced by a similar rate of consumption by antioxidants. Thus, oxidative stress results from the overproduction of ROS in the organism that exceeds the endogenous antioxidant capacity for them to be eliminated. The oxidative and nitrosative stress induced by hyperglycemia is considered one of the primary links between DM and diabetic complications [13]. The mechanism by which hyperglycemia leads to the generation of ROS is primarily due to autooxidation of the glucose and the glycosylation of proteins. The persistent increase in ROS and RNS favors the presence of oxidative and nitrosative stress, with the capacity to produce endothelial dysfunction, insulin resistance, and alterations in the number and functions of the pancreatic *β*-cells, favoring the appearance of micro- and macrovascular complications of DM [14]. The ROS and the RNS cause structural deterioration of the macromolecules (carbohydrates, proteins, lipids, and nucleic acids) causing loss of function [15]. Also, the ROS and the RNS are capable of activating cellular signaling cascades that lead to the transcription of genes that facilitate the development of diabetic complications. The nuclear factor-ĸB (NF-*κ*B) is a nuclear transcription factor that can be activated by the increase in ROS, resulting in the transcription of proinflammatory proteins that aggravate the conditions of the illness. The chemokines and proinflammatory cytokines like the monocyte chemoattractant protein-1 (MCP-1) of the macrophages, the tumor necrosis factor-*α* (TNF-*α*), and the interleukins (interleukin-1*β* and interleukin-6) are implicated in the progression and complications of DM [16]. The increase in ROS and RNS, together with the significant reduction of the antioxidant defense mechanisms in the neurons, contributes to the clinical manifestations of DPN, which include the deterioration of nervous blood flow, endoneurial hypoxia, deterioration of the motor conduction and nerve sensation, degeneration of the peripheral nerves, sensorial loss, axonal atrophy of the large myelinated fibers, and characteristic neuropathic pain [17].

### 3.1. Oxidative Stress of the Endoplasmic Reticulum

The functions of the endoplasmic reticulum (ER) include the protein synthesis and transport, protein folding, lipid biogenesis, maintenance of calcium homeostasis, and the participation of other crucial cellular functions [18]. The ER can control and maintain cellular homeostasis acting as a sensor for stressors in the intra- and extracellular medium, on providing a platform for the interaction between environmental signals and the basic biological cellular functions, and acting as an intersection to integrate multiple responses to stress [19]. The interruption of cellular homeostasis can lead to the gradual reduction in the function of a determinant organ on decreasing the capacity to respond to physiological stress. In fact, it has been suggested that the interruption in ER homeostasis is involved in the pathogenesis of DM and its complications [20]. Study of the behavior of the ER in DPN emerges as an interesting opportunity to investigate the functions of the ER and its signaling network in relation to DM, to develop possible therapeutic strategies [21]. The state of hyperglycemia has the ability to induce oxidative stress of the ER through the accumulation of unfolded or poorly folded proteins into the lumen. When oxidative stress is extreme, or of lengthy duration, the unfolded protein response can be overwhelming, unchaining diverse apoptotic processes including the factor 2 associated with the receptor of the tumor necrosis factor (TNF) and the kinase 1 regulator of the apoptosis signal through activation of the c-Jun N-terminal kinase [22, 23], liberation of calcium from the cytosol, depolarization of the mitochondrial membrane, and the liberation of cytochrome c [24], with excision of the procaspase 12 [25]. Other mechanisms that are altered include the perfusion of nerves [26], the C-peptide release [27], the appearance of dyslipidemia with an increase in the levels of circulating unsaturated fatty acids [28], a decrease in the levels of the glycolysis and intermediaries of the tricarboxylic acid cycle [29], and alterations of the redox state and calcium homeostasis [30]. Furthermore, alterations in the mitochondrial energy metabolism in the neurons of the dorsal root ganglia modulated by the heat shock protein 70 and the ciliary neurotrophic factor are produced [31, 32].

### 3.2. Oxidative Damage to Peripheral Nerves in DPN

The increase in ROS and RNS is capable of causing damage to the lipids present in the myelinated structures of the nerves, resulting in the loss of axons and interruption of the microvasculature in the peripheral nervous system [33]. The oxidative damage to the peripheral nerves causes hyperexcitability in the afferent nociceptors and the central neurons, causing the generation of spontaneous impulses in the axons and the dorsal root ganglia of the nerves, causing neuropathic pain [34].

### 3.3. Oxidative Damage to DNA in DPN

**Figure 2:**
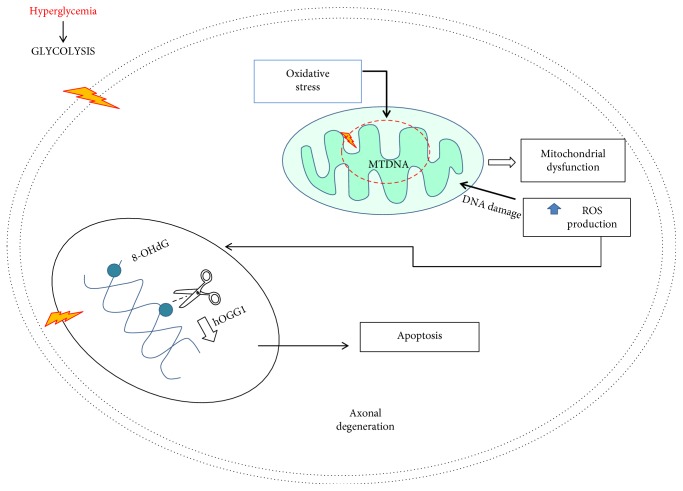
Hypothetical drawing of the effect of hyperglycemia on the increase of oxidative metabolism that induces damage to the mtDNA, which leads to mitochondrial dysfunction. The increase in the production of ROS augments damage to the nDNA with the generation of the product 8-OHdG and the decrease in repair of the DNA in DPN, which can ultimately cause axonal degeneration and cell death.

The oxidative stress that can be produced by the persistent hyperglycemic state in type 2 DM leads to modifications in the mitochondrial genetic material (mtDNA) and the nuclear DNA (nDNA) [35]. The persistence of oxidative damage to the mtDNA is capable of causing mutations in the mitochondrial genome, causing mitochondrial dysfunction that unchains an increase in ROS, forming a vicious cycle within the mitochondria, producing intense oxidative damage that can lead to cell death [36–38]. The mitochondria are the primary source of the production of ROS and RNS and are the first organelles to suffer oxidative damage, putting the cells that are highly susceptible, like neurons, at risk while favoring the progression of DPN. The axons are highly susceptible to the metabolic and endothelial imbalances that lead to the progression of DPN because the axons normally contain a large number of mitochondria. Thus, oxidative damage favors mitochondrial damage of the DNA, mitochondrial dysfunction, and axonal degeneration [39]. On the other hand, H_2_O_2_ has the capacity to move into the cellular nucleus and subsequently the HO• is generated through the Fenton reaction. Then, the reaction of the HO• with the bases of the DNA strand, such as guanine, leads to the generation of radical adducts, then by one electron abstraction, the 8-hydroxy-2′-deoxyguanosine (8-OH-dG) is formed. Other bases of DNA react with HO• in a similar manner; however, the 8-oxodG product is the most abundant and it is relatively easily formed and is promutagenic. In normal conditions, the 8-OHdG can be repaired by the endonuclease 8-oxoguanine DNA glycosylase (hOGG1) enzyme through mechanisms of base excision [40]. However, experimental studies in animals and clinical studies have demonstrated that there are elevated levels of the 8-OHdG marker and deficiency of the DNA repair enzyme in patients with type 2 DM with DPN [41, 42]. The ROS induce activation of the poly(ADP-ribose) polymerase 1 (PARP-1) that undertakes an important role in the repair of damaged DNA through a costly process of energy consumption that causes rapid depletion of the nicotinamide adenine dinucleotide (NAD^+^) with a concomitant decrease in ATP production [43]. Therefore, the control of oxidative damage to the DNA seems to be an important therapeutic target in patients with type 2 DM with DPN (Figure 2()).

In neurons and other cell types, steady-state ATP production is necessary for ion homeostasis, particularly for impulse conduction, as maintenance and post-impulse restoration of the membrane potential are dependent on the activity of Na^+^/K^+^ ATPase. Therefore, ATP depletion elicits sodium to accumulate intracellularly and potassium to diffuse out of the cell causing cell swelling and dilation of the endoplasmic reticulum [44].

## 4. The DAMPs in Diabetes Mellitus

The damage-associated molecular patterns (DAMPs) are intracellularly sequestered molecules involved in the pathogenesis of many human diseases. DAMPs are characterized by being hidden from recognition by the immune system under normal physiological conditions. However, under conditions of stress or cell injury, they may be actively secreted by stressed immune cells or by stress cells in which the neoantigens bind to natural immunoglobulin M (IgM) antibodies. DAMPs can be passively released into the extracellular environment of dying cells or when the extracellular matrix is damaged [45]. DAMPs are recognized by cells of the receptor recognition pattern (PRR) of the innate immune system, including macrophages, leukocytes, dendritic cells, vascular cells, fibroblasts, and epithelial cells for the purpose of promoting proinflammatory and profibrotic pathways [46]. PRRs include RIG-I-like receptors, NOD-like receptors, and Toll-like receptors (TLR) to activate intracellular signaling cascades resulting in the production of cytokines and immunomodulators released from immune cells [47]. In metabolic diseases such as DM, class V DAMPs play a crucial role. This class of DAMPs can be generated by intracellular stress even in nondying cells. This can occur due to minimal metabolic disturbances of homeostasis within the intra/extracellular microenvironment observed in type 2 DM, metabolic syndrome, and obesity. Primary ER disturbances elicit different classes of DAMPs which, through recognition by PRR cells, promote innate inflammation of immune tissue resulting in cell dysfunction. It is essential to consider that metabolism and innate immunity are linked since both systems involve the recognition of exogenous stressors. However, proper management leads to the maintenance of the individual homeostasis of each individual. Recent studies reveal molecular associations between immunity and metabolism because they could be substantial therapeutic targets for sterile inflammatory diseases such as type 2 DM [48]. Therefore, type 2 DM represents the prototype of an innate immune disease where sterile autoinflammatory processes induced by PRR cells trigger dysfunction of *β*-cells and favor cell death (pyroptosis) [49]. It is currently argued that metabolic insults such as insulin resistance, prolonged hyperglycemia, and increased free fatty acids (depletion of calcium levels in the ER) lead to excessive stimulation of insulin production by associated *β*-cells with the accumulation of proinsulin in the ER [50]. Proinsulin overload leads to alterations in ER homeostasis, resulting in accumulation of newly synthesized unfolded or misfolded proteins in the ER lumen that may be considered class V DAMPs [51]. Metabolic disturbances favor ER depletion associated with oxidative stress [52]. The intersection and crosstalk between the innate immune system, stress of ER, and the machinery of the inflammamosome seem to regulate the quality, intensity, and duration of innate proinflammatory and proapoptotic immune responses [53]. It is clear that further studies are required to determine whether the DAMP axis reflects an innate immune pathway that contributes to the pathogenesis of metabolic inflammatory diseases such as DM and its involvement in PND [54].

Mitochondrial DNA (mtDNA) contains a higher frequency of hypomethylated cytosine-phosphate-guanine motifs which are natural ligands for PRR and, therefore, can be recognized by the innate immune system [55]. The mtDNA is highly sensitive to ROS-induced damage, and oxidative stress promoted the fragmentation of mtDNA. It has been shown that after induction of mitochondrial damage by oxidative stress, mtDNA fragments of low molecular weight were released to cytosol via the permeability transition pore [56]. Then, mtDNA fragments can serve as DAMPs when liberated into the extracellular space [57]. Interestingly, mtDNA that escapes from autophagy cell autonomously leads to TLR 9-mediated inflammatory responses. This mechanism might work in inflammation-related diseases such as diabetes mellitus. [58]. In fact, high levels of mtDNA have been reported in peripheral blood mononuclear cells in patients with type 2 diabetes [59] and diabetic retinopathy [60].

## 5. The Role of the Mitochondria in DPN

Mitochondria are the primary source of cellular oxidants, taking into account that about 2% of molecular oxygen is not completely reduced to water at the electron transport chain and, therefore, is the primary site for the potential overproduction of ROS and a prime target of cumulative oxidative damage. The mitochondria play a critical role in the regulation of the metabolic imbalance observed in DM, since both H_2_O_2_ and ONOO− can cross the mitochondrial membranes and damage macromolecules in other cellular regions [61]. An increase in the levels of O_2_•− in the mitochondrial electron transport chain as a result of the hyperglycemic state that favors the increase of oxidative stress has been reported [62]. Other metabolic pathways involved in ROS production, which augments the oxidative stress in DM, are the synthesis of metabolites through the xanthine oxidase pathway, the production of neurotransmitters, and the detoxification of the xenobiotics through the cytochrome P450 system and the NADPH oxidase [63].

**Figure 3:**
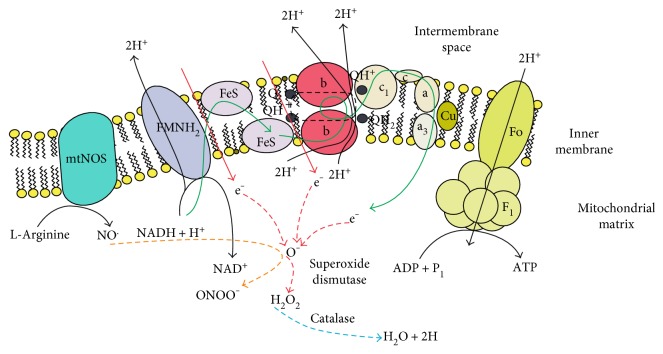
Formation of reactive oxygen and nitrogen species in mitochondria. The process is mediated by oxidative phosphorylation and the activity of the mitochondrial NO synthase: in physiological conditions the production of ROS and RNS are reduced by multiple steps that involved SOD, GPx and catalase. When the mitochondria suffers an insult the increase of the leakage of electrons to the matrix leads to an overload to the capacity of the enzymatic systems and leads to toxicity of the cell. Black arrows: vectors of reactions and products. Green arrows: the physiological pathway for formation of oxidative stress. Red arrows: leakage of electron to matrix. Dotted red and orange arrows: pathophysiological pathway for formation of ROS and RNS.

Because diabetic cells exhibit high glucose content, excess of glucose-derived pyruvate is oxidized through the tricarboxylic acid cycle which causes higher levels of electron donors (NADH and FADH_2_) to the electron transport chain. This exceeds the capacity of the electron transport chain and blocks the electron transfer in the ubiquinol-cytochrome c reductase complex, causing the electrons to return to coenzyme Q. Thus, an increasing level of O_2_•− is observed. O_2_•− is a relatively small anion; in fact, the hydration shell of the superoxide anion is relatively small, with only four protons being strongly coupled to the unpaired electrons. The superoxide dismutase (SOD) enzyme degrades this oxygen-free radical to H_2_O_2_, which is then converted to H_2_O and O_2_ by other enzymes such as catalase and glutathione peroxidase [64] (Figure 3()). H_2_O_2_ affects lipids and intramembranous proteins. It is a ROS whose biological actions are governed by its chemical reactivity towards biological targets, among which are metalloenzymes such as hemoperoxidases and amino acid residues sensitive to oxidants such as cysteine [65].

HO• is actively involved in lipid peroxidation and is associated with the genesis of harmful factors involved in many chronic degenerative diseases [66]. HO• can attack macromolecules (lipids, nucleic acids, and amino acids). Phenylalanine can be converted enzymatically into a physiological para-tyrosine. The attack of HO• on phenylalanine can produce para-tyrosine, meta-tyrosine, and ortho-tyrosine. The target and ortho-tyrosine are considered markers of HO•-induced damage. The use of resveratrol to treat patients with type 2 DM leads to decreased urinary excretion of ortho-tyrosine and concomitantly improves insulin signaling and sensitivity to this hormone [67]. Thus, the administration of resveratrol may be an attractive therapeutic tool along with strict metabolic control in patients with DM and chronic complications of DM.

## 6. Nitric Oxide

The production of •NO occurs from the L-arginine by the nitric oxide synthase (NOS) (Figure 3). The NOS has four isoforms: neuronal (nNOS), inducible (iNOS), endothelial (eNOS), and mitochondrial (mtNOS) [68]. The •NO is implicated in physiological processes like vasodilation, the modulation of nociception, the immune function, neurotransmission, and the excitation-contraction coupling [69]. The •NO is considered an atypical neurotransmitter and a second messenger in the nervous system [70] or as a hormone [71]. The majority of the effects of •NO are mediated through activation of the guanylate-cyclase enzyme that produces cyclic guanosine-3,5-monophosphate (cGMP) [29]. The •NO has pronociceptor properties in the neural crest and in the dorsal root ganglia that positively regulate as a result of cutaneous or visceral inflammation and by the peripheral lesions of the fibers. This effect could be potentiated or inhibited by the •NO donors [72].

O_2_•− interacts with •NO, forming the potent ONOO− that attacks several biomolecules, conditioning the production of a modified amino acid: nitrotyrosine [73]. Nitrotyrosine was initially considered a specific marker of ONOO− generation, but other pathways such as mieloperoxidase may also induce nitrosation of tyrosine. Nitrotyrosine is often described as a stable marker of oxidative/nitrosative stress [74]. Nitrosative stress-induced damage plays a crucial role in multiple interrelated aspects of the pathogenesis of DM and its complications. In the state of hyperglycemia, it stimulates the production of ONOO− capable of damaging the vascular endothelium and the peri-neuro in DPN [75]. Angiotensin II is also capable of inducing intraendothelial ONOO− production and activation of poly(ADP-ribose) polymerase (PARP) [76]. Angiotensin II is capable of inducing direct prooxidant effects on the vascular endothelium. The effects of angiotensin II are mediated in part by the formation of intraendothelial ROS through the family of nonphagocytic NAD(P)H oxidase subunits. ROS produced after angiotensin II-mediated stimulation have the ability to exert direct oxidative effects through pathways such as mitogen-activated protein kinases, tyrosine kinases, and transcription factors that promote inflammation, hypertrophy, remodeling, and angiogenesis [77]. Inhibition of angiotensin II by the angiotensin-converting enzyme (ACE) *in vivo* seems to reduce the formation of ONOO− [78]. Neutralization of RNS or inhibition of PARP activation pathways may emerge as a new approach, first as experimental therapy of DM, even for the prevention or reversal of complications caused by DM.

## 7. Mitochondrial Dysfunction in DPN

Mitochondria are the major sites of adenosine triphosphate (ATP) synthesis by the processes of oxidative phosphorylation. Mitochondria also mediate amino acid biosynthesis, fatty acid oxidation, steroid metabolism, calcium homeostasis, and ROS production and detoxification. Often, the mitochondria accumulate in the synapses and play a predominant role in synapse maintenance through attenuation of the Ca^2+^. A lot of neurons depend on the mitochondria, and so there is a strong link between neuronal dysfunction and mitochondrial dysfunction [79]. The indicators of mitochondrial dysfunction present in neurodegenerative illnesses include ultrastructural changes, inhibition of the respiratory chain, decrease in ATP production, an increase in the production of FR, deletions of the mtDNA, loss of calcium buffer effect, and loss of the mitochondrial membrane potential [80]. Mitochondria are dynamic bodies that constantly divide and fuse within the cell as the environment demands [81]. These processes can facilitate formation of new mitochondria, repair of defective mitochondrial DNA through mixing, and redistribution of mitochondria to sites requiring high-energy production [82]. Both processes effectively lower the percentage of defective mitochondria in the cell and ensure stability in cellular proliferation; indeed, metabolism, energy production, calcium signaling, reactive oxidative species production, apoptosis, and senescence all depend on the balance of fission and fusion. Conversely, dynamic distortion (i.e., excessive fragmentation/elongation) results in inefficiencies in cell functioning, if not cell death [83, 84]. Mitochondrial dynamics is a tightly regulated cellular process, with sophisticated molecular machinery involving GTPases. Fission is regulated by at least two proteins: a large GTPase, dynamin-like protein 1 (Drp1), and a small molecule, Fis1, and fusion involves three large mitochondrial transmembrane proteins localized to the outer membrane: mitofusin 1 (Mfn1), mitofusin 2 (Mfn2), and optic atrophy protein 1 [82, 85]. One model of mitochondrial fission suggests that the Drp1 is formed into rings or spirals that surround the external mitochondrial membrane with the help of hFis1 and other cofactors and regulators yet to be discovered. It is thought that GTP hydrolysis causes a conformational change in Drp1 that drives the fission event of the external mitochondrial membrane [86]. The excess of mitochondrial fission is an early and important event in neurodegenerative illnesses. The oxidative and nitrosative stress appear to play a predominant role as inductors of mitochondrial fission [87]. Several studies suggest that the damage to DNA and hyperglycemia can stimulate mitochondrial fission and indicate that the aberrant activation of components of the cellular cycle in postmitotic neurons plays an important role in the regulation of the mechanics of mitochondrial fission [88, 89]. Damage to the DNA is an event that can unchain mitochondrial fission, which can contribute to neuronal loss [90]. Mitochondrial fusion requires components of the external and internal membrane. Mfn1 and Mfn2 facilitate fusion of the external membrane in mammals, probably through transinteractions that promote the curvature and fusion of the membrane [91]. Some studies suggest that the GTPase is the principle mediator of fusion of the internal membrane and of the maintenance of the mtDNA in mammals. The mutations in the proteins of mitochondrial fusion give way to greater mitochondrial fragmentation, which could favor the appearance of neurodegenerative illnesses such as Parkinson's, Alzheimer's, and Huntington's diseases, among others [92, 93]. The dorsal root ganglion neurons (DRGs) exposed to hyperglycemia present with mitochondrial dysfunction, fragmented mitochondria, and an increase in the expression of Drp1 and oxidative stress [94]. Hyperglycemia stimulates an increase of the Drp1/Bax complexes, which mediate apoptotic mitochondrial fragmentation [95].

## 8. Autophagy

Autophagy is a catabolic process induced by oxidative stress that involves the delivery of cytoplasmic materials to the lysosome for degradation and component recycling. It is considered a protector of the cells against diverse factors of stress and routine wear and tear and is characterized by the sequestration of organelles/senescent or damaged proteins, forming autophagosomes to recycle those products [96]. Autophagy is involved in the elimination of cells that have suffered programmed cell death type 1 (classic) and in one form of nonapoptotic cell death or cell death type 2. Therefore, autophagy protects the cells on promoting cell death, depending on the state and cellular environment in which they are found [97]. The increase in ROS is essential for autophagy to prosper because in the presence of ROS it is possible to control the Atg4 activity, a family of cysteine proteases that are necessary for the formation of autophagosomes [98]. Autophagy can be inhibited by the regulator protein the mammalian target of rapamycin (mTOR) [99]. Recently, it was reported that the activation of PARP-1 induced by the ROS promotes autophagy through the activation of AMP-activated protein kinase (AMPK), likely by suppression of the mTOR [43]. The deregulation of autophagy is related to pathologies like cancer, myopathies, neurodegenerative illnesses, heart diseases, liver diseases, gastrointestinal disturbances, and the complications of DM [100]. Autophagy can be categorized in three classes: macroautophagy, chaperone-mediated autophagy (CMA), and microautophagy [101]. The primary focus of the macroautophagy involves the formation of autophagosomes (double-membrane vesicles) in a multistep process. The autophagosomes combine with the liposomes and degrade the content through diverse acid hydrolases. This process is mediated by more than 30 autophagy-related proteins (Atg). Macroautophagy consists of two subsets: autophagy of specific organelles and selective macroautophagy. Although substantial progress has been made in the understanding of the complex mechanisms that regulate autophagy, many interactions involved in the control of the process have not yet been adequately described [102]. The ROS inhibit the activity of the mTOR signaling protein on invoking the dephosphorylation of the Atg13, the activation of the serine/threonine protein kinase ULK, and the recruitment of the focal adhesion kinase family-interacting protein of 200 kD (FIP200). The ULK-Atg13-FIP200 complex plays a critical role in the formation of autophagic double-membrane vacuoles in forming autophagosomes capable of disposing cellular waste. Microautophagy has been discussed little in chronic degenerative diseases [43].

### 8.1. Autophagy in DPN

Numerous metabolic and cellular alterations in neural tissue because of DM have been described, including the state of dyslipidemia, the excessive generation of ROS and RNS, and, obviously, the state of hyperglycemia [103]. These alterations cause mitochondrial and cytosolic oxidative stress with the generation of abnormal glycated proteins and dysfunctional mitochondrial proteins [104]. These alterations are a growing field of research which suggests that autophagy occurs as a cytoprotective response [105]. Autophagy in neural tissue has been described as a mechanism of cleansing that eliminates the damage caused by cellular stressors [106]. Mounting evidence shows that autophagy plays a potentially significant role in the pathophysiology of DPN, which requires additional research to completely understand the mechanisms that unchain the induction of autophagy in the nerves of diabetics and the relationship with neuronal injury during the natural history of DPN. Still pending to answer are numerous questions with regard to the relative contribution of the different stress factors in the process of autophagy and the cascade of interactions of autophagy with other cellular signals [66].

Rapamycin, an immunosuppressive drug that induces autophagy, has the ability to affect other aspects of cellular function, and it could be a focus of therapeutic interest in DM and its complications [107]. It has been reported that rapamycin improves tolerance to glucose in experimental animals fed with a diet rich in fats supplemented with branched chain amino acids, but not with high fat diets [108], which suggests the possible role of rapamycin or rapamycin-related compounds in type 2 DM [109]. Thus, the development of treatments that favor the cytoprotective effect of autophagy in the complications of DM is a potentially promising research path.

## 9. Management Alternatives in DPN

Currently, an absolute cure has not been defined for DPN or any other complications of DM, although some medications are conventionally useful. However, it is interesting to consider the pathophysiological links between hyperglycemia and oxidative stress. As well, the superior adjuvant effect of the antioxidants and FR scavengers continues to be essential in the prevention of DPN in diabetic patients.

### 9.1. Glycemia Control

Achieving control of stable glycemia is the only, most effective, and most difficult goal to achieve therapeutic target for the management of the complications of DM. According to reports from the follow-up study, “Diabetes Control and Complications and Epidemiology of Diabetes Interventions and Complications” (EDIC), the intensive glycemic control designed to achieve nearly normal blood glucose levels, implemented early in the course of the diabetes, delays the development of DPN in patients with type 1 DM, without obtaining the same results in type 2 DM [110]. To date, an absolute cure for DN has not been defined. Although some drugs are conventionally used, some may be found in which some aspects of the pathophysiological links with oxidative stress are known.

### 9.2. Antioxidants

Antioxidants diminish or delay the oxidation of other molecules by inhibiting the initiation or propagation of oxidizing chain reactions, thus reducing its capacity to damage. Antioxidants may act as radical scavengers, peroxide decomposers, hydrogen donors, electron donors, singlet oxygen quenchers, enzyme inhibitors, or metal-chelating agents [111]. Their effect depends on concentration [112], polarity, and the medium [113], and also the presence of other antioxidants [114]. In fact, antioxidants may act from directly scavenging free radicals to increasing antioxidative defenses. There are several types of antioxidants in cells: dietary antioxidants (vitamins A, C, and E), endogenous antioxidant enzymes (superoxide dismutase (SOD), catalase, glutathione peroxidase, glutathione reductase (GPx), glutathione S-transferase (GST), and peroxiredoxins), and antioxidant molecules (glutathione (GSH), coenzyme Q, ferritin, bilirubin, uric acid, lipoic acid, melatonin, carotenoids, and flavonoids). Under physiological conditions, these molecules and enzymes work synergistically and together with each other to protect the cells [115]. The SOD dismutates the O_2_•− to form H_2_O_2_ upon which acts as the catalase or the GPx to produce water. The GST converts the reactive electrophilic species to form easily excretable hydrophilic products as a result of the conjugation with GSH. Vitamins C and E and the alpha-lipoic acid are involved in the elimination of the products of lipoperoxidation (LPO) [116]. As well, the flavonoids are capable of eliminating FR [117]. Some specialized proteins have regulator functions of the redox signaling with an antioxidant effect, like the peroxiredoxins (Pxr), thioredoxins (Trx), and glutaredoxins (Grx), with intracellular effects on the ROS and RNS [118]. The members of these families of proteins are ubiquitously expressed in all organisms, tissues, types of cells, and organelles. Some of these proteins can also move between cellular compartments and the extracellular space [119].

The stoichiometric number of antioxidants that capture FR by an antioxidant molecule and the effectiveness of FR scavenging can be evaluated by performing *in vitro* tests. The biological functions of antioxidants have been widely evaluated for their effects on the expression of antioxidant enzymes. For example, *γ*-tocopherol is a relatively mild ROS scavenger when compared to *α*-tocopherol. However, the oxidized product, *γ*-tocopheryl-quinone, reacts readily with the thiols to release the nuclear factor (Nrf-2) resulting in the expression of antioxidant enzymes such as hemooxygenase-1 [120].

There are several existing strategies with the use of different antioxidants to manage DPN. The choice of antioxidant depends on its chemical structure and concentration, the type of DPN, and stage of the illness, its severity, and the prevalence and primary causes from which it originated [121]. The antioxidants have different mechanisms and action sites through which they exert their biochemical effects and improve nerve dysfunction produced by oxidative stress in DPN.

### 9.3. Metformin

Metformin is a widely prescribed oral antidiabetic agent that reduces the production of hepatic glucose and improves peripheral sensitivity to insulin. The antihyperglycemic mechanisms of action of metformin include decrease of the absorption of glucose by the small intestine, increase in glucose uptake by the cells, decrease in concentrations of fatty acids free in plasma, and inhibition of gluconeogenesis through the activation of protein kinase activated by the AMP (AMPK). Other mechanisms of action of metformin are related to its antiatherosclerotic action, hypotensive and anticarcinogenic action, and its impact on the endothelial function in the veins. The pleiotropic actions of metformin include the impact on plasma lipid profiles, the decrease of oxidative stress, and the increase in the fibrinolytic activity in the plasma. Metformin is actively transported to the hepatocytes and the renal tubular epithelium by organic cation transporters 1 and 2 coded by the corresponding SLC22A1 and SLC22A2 genes, respectively. The transporter of the multi-antimicrobial extrusion protein 1 (MATE1) coded by the gene SLC47A1 facilitates the excretion of metformin through bile and urine [122]. It seems that metformin reduces the accumulation of autophagic vesicles and death of the pancreatic *β*-cells in patients with type 2 DM. These effects can be associated with the restored expression of the lysosome membrane-associated protein-2 [123]. Metformin is capable of inhibiting the mTOR pathway independently of the AMPK, and it promotes the generation of and elimination of autophagic vesicles [124].

### 9.4. Vitamins

The antioxidant vitamins A, C, and E are ingested with food and are capable of directly neutralizing and detoxifying FR and interacting with the recycling processes to create reduced forms of the vitamins [125]. The antioxidant vitamins have diverse biological activities in stimulating the immune system and preventing genetic changes through inhibiting the oxidative damage to DNA [126]. There is little information on the role of vitamin C in DPN, though there is evidence that it normalizes the concentration of sorbitol in blood and diminishes LPO and regenerates GSH in DM [127].

Vitamin E or tocopherols react with the OH• to form a stable phenolic radical that is reduced to a phenol by the ascorbate and the NADPH enzyme dependent on the reductase enzymes [70]. Vitamin E has a preventative effect in diabetic complications through the decrease in LPO, although without demonstrating significant improvement in symptomatology of the micro- and macrovascular complications despite reducing the markers of oxidative stress [128]. Vitamin E output is directed toward DPN because it has the ability to reduce neuropathic pain through modulating oxidative stress in the dorsal root ganglia [129]. Supplementation with vitamin E has been reported to significantly reduce blood glucose levels and glycated hemoglobin and has a neuroprotector effect in the myenteric nerves without affecting the intestinal area, the thickness of the intestinal wall, or muscular tone [130, 131].

L-Methylfolate, the active form of folic acid, is 7 times more bioavailable than folate and 3 times more effective in reducing homocysteine levels than folic acid [132]. Although the role of folic acid in vascular disease is not well established, active folate (5-methyltetrahydrofolate) can regenerate tetrahydrobiopterin (BH4). L-Methylfolate plays a role as an enzymatic cofactor for the conversion of the guanidinium nitrogen of L-arginine (L-Arg) to NO [133].

Benfotiamine is a synthetic lipid form of thiamine (B1) developed in Japan in the late 1950s to treat alcoholic neuropathy, sciatica, and other painful nerve conditions. Benfotiamine increases intracellular levels of thiamine di-phosphate (transketolase cofactor). This enzyme reduces AGE and LPO by directing its substrates to the pentose phosphate pathway. Reduction of AGE has been shown to contribute to the prevention of macro- and microvascular endothelial dysfunction in individuals with type 2 DM. Prospective cohort studies involving folic acid, benfotiamine, and its metabolites in PND patients will yield interesting results [134].

### 9.5. Flavonoids

**Figure 4:**
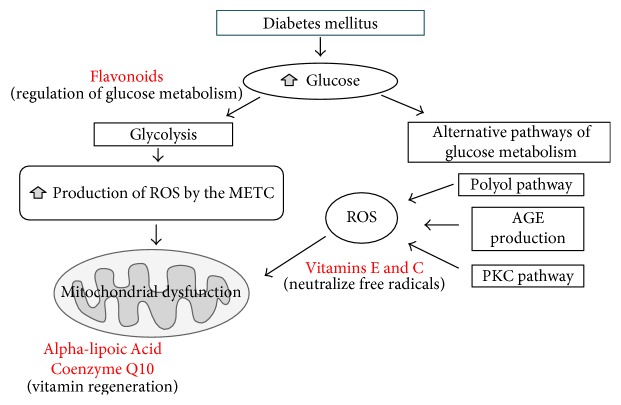
We show the theoretical mechanism of how hyperglycemia favors the activation of several metabolic pathways that favor the production of ROS causing mitochondrial dysfunction. The beneficial action of antioxidants in the regeneration of antioxidant vitamins and the effect of flavonoids in the regulation of hyperglycemia.

The flavonoids are the largest and most important group of polyphenolic compounds. The flavonoids are widely distributed in plants, fruits, vegetables, grains, roots, stems, flowers, tea, and wine [135]. The antidiabetic properties of the flavonoids are primarily based on their effect on diverse molecular objectives and in the regulation of various pathways, like the reduction of apoptosis, improvement of proliferation of the pancreatic *β*-cells, promoting the secretion of insulin through regulation of glucose metabolism in hepatocytes on improving hyperglycemia, and decreasing insulin resistance, inflammation, and oxidative stress in adipocytes and skeletal myofibrils. They also favor the uptake of glucose by the skeletal muscle and adipose tissue [136]. Some subclasses of flavonoids can eliminate FR and chelate metals [137]. Taurine, acetyl-L-carnitine, and acetylcysteine have also reportedly demonstrated reducing the progression of DPN [15]. Polyphenols are potent antioxidants capable of contributing to the prevention of type 2 DM through its anti-inflammatory, antimicrobial, and immunomodulating properties. Citrus fruits contain polyphenols that have antioxidant and antidiabetic activity. Citrus polyphenols are mainly contained in the shell and have the ability to capture free radicals, in addition to antioxidant activity [138] (Figure 4).

### 9.6. Aldose Reductase Inhibitors

Inhibitors of aldose reductase in humans belong to the superfamily of aldo-keto-reductase proteins, characterized by catalyzing and limiting the polyol pathway of glucose metabolism by reducing glucose to sorbitol. Inhibitors of aldose reductase also reduce a wide range of aldehydes by detoxifying toxic lipids generated by oxidative stress by combining them with glutathione [139]. Accelerated flow of sorbitol through the polyol pathway has been implicated in the pathogenesis of secondary diabetic complications such as PND [140]. Previously, it was reported that the administration of albinase reductase inhibitors sorbinil or fidarestat in diabetic rats was able to correct depletion of glutathione and ascorbate induced by DM. At the same time, they are capable of correcting the negative regulation of SOD enzyme activity and the accumulation of LPO products in the peripheral nerves of the formation of O_2_•− *vasa nervorum* of the retina associated with oxidative and nitrosative stress with the ability to inhibit the accumulation of poly(ADP-ribose), a marker of PARP activation in the diabetic nerve and retina [141]. Although in experimental animals, aldose reductase inhibitors have demonstrated the potential inhibition of secondary diabetic complications, none of the aldose reductase inhibitors have been subjected to phase III clinical trials for the prevention of PND [142]. Recent studies suggest that increasing the polyol pathway could alter the NADPH/NADP ratio and attenuate GPx the GR by decreasing the reduced glutathione/oxidized glutathione which would cause oxidative stress [143]; it is interesting to explore the role of inhibition of aldose reductase in PND patients with a minimum follow-up of 5 years.

### 9.7. Free Radical Scavengers

#### 9.7.1. Alpha-Lipoic Acid

Alpha-lipoic acid is a hydrophilic and lipophilic acid that can be synthesized by plants and animals where it is metabolized to dihydrolipoic acid when captured by the cells [144]. The alpha-lipoic acid and the dihydrolipoic acid are potent eliminators of FR and are involved in the regeneration of vitamins C and E and the GSH in the cell. Alpha-lipoic acid is also a cofactor for the production of diverse mitochondrial enzymes [145]. The ingestion of alpha-lipoic acid at a dose of 200–600 mg can provide up to 1000 times the quantity of available alpha-lipoic acid present in a regular diet. Preclinical and clinical data indicate that alpha-lipoic acid is safe and can be bioavailable in moderate doses. Gastrointestinal absorption of alpha-lipoic acid is variable and requires the consumption of food: its intake is recommended 30–60 min before food or 120 min after a meal [76]. It is rapidly absorbed and reaches maximum levels in the blood in 30–60 min, with a parenteral half-life of 30 min [146], and its consumption is considered safe in liver and kidney diseases [147]. One study done with alpha-lipoic acid over a four-year period reported that it is well tolerated in mild-moderate DPN and demonstrated significant clinical improvement and prevented the progression of the neuropathic disturbances without, at the end, impacting improvements in the neurophysiologic tests [148].

#### 9.7.2. Resveratrol

Resveratrol is a stilbene compound and a phytoalexin. It is abundantly present in red wine, berries, red grapes, blueberries, peanuts, teasadori, hops, pistachios and grape juice, and cranberry. The antihyperglycemic effects of resveratrol appear to be the result of increased action of the glucose transporter on the cytoplasmic membrane. Neurons are extremely susceptible to damage induced by oxidative stress due to their high rate of oxygen consumption and low levels of antioxidant defense enzymes. The protective actions of resveratrol in DPN are attributed to its intrinsic FR scavenger properties. However, many other associated or separate mechanisms have recently been proposed such as the upregulation of Nrf2, SIRT1, and inhibition of the NF-*κ*B transcriptional factor with a beneficial effect against nerve dysfunction [149]. Resveratrol emerges as an interesting management alternative to glycemic control in patients suffering from DPN.

## 10. In conclusion

The fundamental characteristic of patients with DPN is hyperglycemia with the capacity to unchain multiple and diverse processes among which are oxidative stress, RE oxidative stress, oxidative damage to DNA, mitochondrial dysfunction, alterations in the physiology of autophagy, the deregulation of endogenous antioxidants, and the variable effect of exogenous antioxidants in relation to metabolic control. Oxidative stress induced by hyperglycemia is mediated by several widely identified traditional signaling pathways, which at the same time are interesting therapeutic targets: (a) polyol, (b) hexosamine, (c) protein kinase C, (d) advanced glycosylation end-products, and (e) glycolysis. Management alternatives of these alterations emerge as interesting therapeutic targets in the study of the mechanisms of action at the molecular level as the FR scavengers and some nutrients with an antioxidant effect, always trying to correct the state of hyperglycemia.
